# Delayed self-regulation and time-dependent chemical drive leads to novel states in epigenetic landscapes

**DOI:** 10.1098/rsif.2014.0706

**Published:** 2014-11-06

**Authors:** Mithun K. Mitra, Paul R. Taylor, Chris J. Hutchison, T. C. B. McLeish, Buddhapriya Chakrabarti

**Affiliations:** 1 Department of Physics, I.I.T. Bombay, Mumbai 400076, India; 2 Systems Biology Doctoral Training Centre, University of Oxford, Oxford OX1 3QU, UK; 3 School of Biological and Biomedical Sciences, Durham University, Durham DH1 3LE, UK; 4 Department of Physics and Astronomy, Durham University, Durham DH1 3LE, UK; 5 Department of Mathematical Sciences, Durham University, Durham DH1 3LE, UK; 6 The Isaac Newton Institute of Mathematical Sciences, University of Cambridge, Cambridge CB3 0EH, UK

**Keywords:** epigenetics, mathematical modelling, gene regulatory networks

## Abstract

The epigenetic pathway of a cell as it differentiates from a stem cell state to a mature lineage-committed one has been historically understood in terms of Waddington's landscape, consisting of hills and valleys. The smooth top and valley-strewn bottom of the hill represent their undifferentiated and differentiated states, respectively. Although mathematical ideas rooted in nonlinear dynamics and bifurcation theory have been used to quantify this picture, the importance of time delays arising from multistep chemical reactions or cellular shape transformations have been ignored so far. We argue that this feature is crucial in understanding cell differentiation and explore the role of time delay in a model of a single-gene regulatory circuit. We show that the interplay of time-dependent drive and delay introduces a new regime where the system shows sustained oscillations between the two admissible steady states. We interpret these results in the light of recent perplexing experiments on inducing the pluripotent state in mouse somatic cells. We also comment on how such an oscillatory state can provide a framework for understanding more general feedback circuits in cell development.

## 1. Introduction

The ‘biological impossibility’ of reprogramming adult somatic cells to the pluripotent state had been accepted as a dogma for a long time in biology [1]. This view was radically changed by the work of John B. Gurdon in 1962, who showed that a nucleus from a fully differentiated frog intestinal epithelial cell could generate a functioning tadpole upon transplantation into an enucleated egg [2,3]. In another seminal work, Shinya Yamanaka and co-workers demonstrated for the first time in 2006, that four transcription factors (Sox4, Oct2, Klf-4 and c-Myc) were capable of reprogramming an adult mouse fibroblast cell to pluripotency [4]. These induced pluripotent stem cells (iPSCs) were fully germline-competent and were used to clone fully functioning adult mice [5–7]. The discovery of germline-competent iPSCs has opened up a new avenue for understanding the process of cellular differentiation besides offering a new source for developing stem cells for tissue regeneration and other biomedical applications, without the ethical concerns of harvesting embryonic stem cells. Transcription factor-based somatic cell reprogramming has since been shown to be a robust process, and human pluripotent cells have also been developed from somatic cells using a combination of transcription factors, using the SOKM protocol [5] as well as using other TFs such as NANOG and Lin28 in place of Klf-4 and c-Myc [8,9]. While induced pluripotency has been characterized for a number of different cell lines, understanding the key gene regulatory networks and molecular mechanisms that underlie the process remains a key outstanding challenge [10–12].

Cell development and differentiation has been interpreted in the light of Waddington's epigenetic landscape [13], visualized as a set of marbles rolling down a hill with the position of the marble indicative of the state of cellular development. Thus, undifferentiated cells all start at the same state at the top of the hill and end up in different valleys corresponding to their differentiated states at the bottom of the hill depending on the surface topography. These differentiated cell states are separated by barriers which prohibit their spontaneous transformation from one state to another. Though visually compelling and despite past attempts a quantification of Waddington's landscape has been attempted only recently [14–17].

Cell developmental circuits have been modelled as self-regulatory networks, where a transcription factor promotes its own production [14–17] as well as inhibits the production of other TFs (in multi-variable models) [14]. Such TF-regulated gene networks are known to accurately represent cell fate decision pathways in biological models. A two variable self-activating and mutually inhibiting gene network has been found in various tissues, where a multipotent cell undergoes a binary decision process [14,18,19]. One known instance is when the common myleoic progenitor differentiates into either the myeloid or the erythroid fate, depending on the expression levels of the PU.1 and the GATA1 transcription factors [14,19,20]. Such models have been useful in providing a quantitative description of developmental landscapes that correspond to the spirit of Waddington's landscape, with different basins of attraction representing the valleys of the differentiated states.

An important aspect of the reprogramming process is identifying the pathways through which a fully differentiated somatic cell is programmed back to pluripotency, and in particular, whether the path a cell takes in going from a somatic state to a pluripotent state is the same as the reverse pathway. Also of interest is characterizing the possible intermediate states in the process. Recent experiments by Nagy & Nagy [10] have shed some light on the path the cell takes as it is reprogrammed back to a pluripotent state. They studied the reprogramming of differentiated secondary mouse fibroblast cells that were derived from iPSCs and encoded the four Yamanaka factors under the control of doxycycline promoters. Thus, expression of the four factors and induction of pluripotency in entire populations of the fibroblasts could be achieved by treating cultures with the drug doxycycline. They found that there were two distinct timescales in the reprogramming process, a point of no-return (PNR) time and a commitment to pluripotent state (CPS) time. The conversion of the cell from the somatic state to the pluripotent state is a slow process, and it takes about 21 days for the somatic cell to reach pluripotency under the effect of the doxycycline input. There are numerous changes associated with the return to a pluripotent state, and the external drive (doxycycline) input needs to be provided for a time of about 14 days for the endogenous factors to become active and drive the cell to pluripotency in the absence of the doxycycline input. This time is called the CPS timescale. Similarly, the PNR timescale, at about 7 days, indicates the time below which the cell returns to the somatic state if the external doxycycline input is removed. The biological changes associated with the two timescales are not clearly understood and require further experiments to clarify. In between these two timescales, the PNR and the CPS, they found that the cell reached an undetermined state, which was neither somatic nor pluripotent, but rather signals the presence of a novel intermediate state in the reprogramming process. Cessation of the doxycycline input during this period results in neither return to somatic nor progress to pluripotent states. They denoted this novel intermediate state as the ‘Area 51’ state. However, the characteristics of this state have not yet been determined.

The presence of an intermediate state in the reprogramming pathway promises to be a useful tool in understanding the mechanics of the uphill process. Furthermore, a full understanding of the Area 51 state could lead to enhanced control over the reprogramming process, such as offering the possibility to create and maintain lineage-committed cells that have various applications. In this paper, we propose a theoretical framework that can lead to such intermediate states in the context of a gene regulatory network. Our work focuses on deterministic approaches to modelling the gene regulatory network, in which the system attains a steady state depending on the choice of parameters, and stays in the steady state once it is reached. In biological systems, the cell may switch between different steady states, and this can be modelled by introducing stochastic dynamics into the model, in which fluctuations may lead to transitions between attractors [21]. While this deterministic differential equation approach is an abstraction of an inherently discrete and stochastic process, it has been shown to be a powerful tool on analysing gene regulatory networks and has yielded experimentally verifiable predictions for a large number of systems. Since the epigenetic reprogramming process is characterized by an overexpression of the associated transcription factors (Sox, Oct-4, Klf-4 and c-Myc), which drives a somatic cell deterministically to the induced pluripotent cell fate, it is expected that a deterministic approach provides a reasonable modelling paradigm for the epigenetic landscape. In this paper, we focus on the deterministic gene networks, and the study of the effect of stochastic fluctuations is left for future work. A comparative analysis of deterministic and stochastic approaches to modelling gene regulatory networks can be found in [22,23].

The reprogramming of a somatic cell to pluripotency is a complex multistep reaction that involves both structural modifications to the chromatin network as well as changes in gene expression patterns [24,25]. These changes arise in response to the expression levels in the gene regulatory network and are modelled by a self-regulating feedback loop. However, since these changes occur in a finite time, the feedback loop should in fact depend on the state of the system at a previous instant of time, leading to delays. Delay differential equations have been used to study diverse systems [26], such as modelling disease onset in physiological systems [27] and discrete time population models [28]. Biochemical circuits involving feedback and delay have also been studied and the general criterion for oscillations to exist in such systems, i.e. existence of a (i) delayed negative feedback, (ii) nonlinearity in the chemical kinetics, and (iii) a proper balancing of timescales for forward and backward reactions, identified [29]. These studies (see [29] and references therein) focused on delayed negative feedback with nonlinear chemical kinetics in the degradation term that renders the steady-state ‘unstable’ leading to oscillations for a choice of model parameters. In this work, we show that a time-dependent chemical drive and a delayed positive feedback with no delay on the degradation term of a chemical reaction leads to oscillations for certain choices of delay parameters. We show that this interplay between a time-dependent drive and a delayed positive feedback is critical in developing a mathematical framework for understanding the nature of the epigenetic landscape.

In this paper, we model the epigenetic landscape through the dynamics of a single differentiation regulator, denoted by *x*, that promotes its own synthesis through a feedback loop. While real-life regulatory circuits in the cell depend on two or more differentiation regulators, the main aim of this paper is to show the effects of time delays in such circuits, and a single-variable genetic circuit offers a model system in which to study such effects. Such single-variable circuits are similar to the models proposed for progesterone-induced *Xenopus* oocyte maturation [15–17,30,31] and might also be applicable to scenarios where a single transcription factor such as MyoD has been shown to induce a change of cell fate from fibroblast to myoblast [32]. We define the single-variable regulatory model in the next section and discuss the results as a function of the parameters of the model. A discussion of the importance and applicability of the resulting phase diagram to systems of differentiating cells and its extension to more realistic gene regulatory networks are discussed in §3.

## 2. Model and results

Gene regulatory networks that control cell fate differentiation have been modelled by self-activating genes. While actual gene regulatory networks inside the cell may consist of multiple genes which have a complex interdependence on each other, one or two-variable gene networks provide a useful model to illustrate some of the basic principles of cell fate determination.

**Figure 1.**
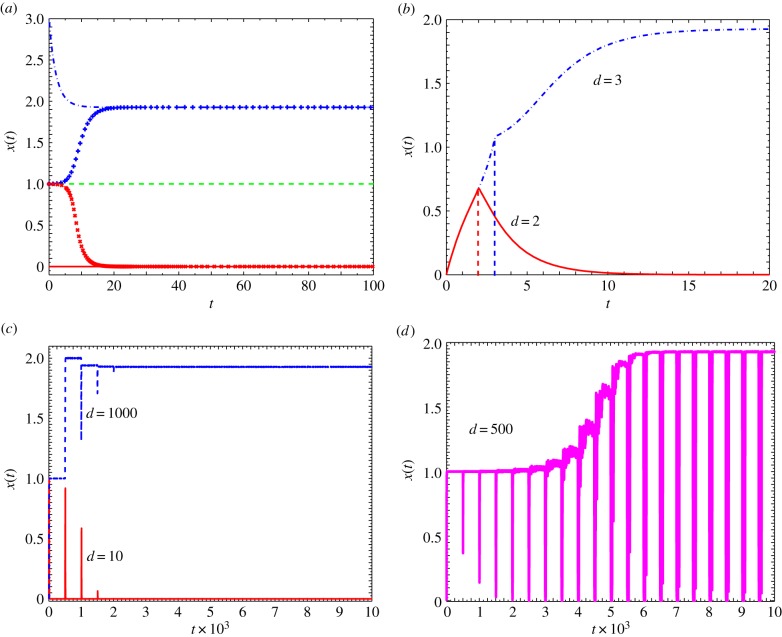
Cell differentiation in single-gene regulatory network with delay. Somatic (*x* = 0), induced pluripotent (*x* ≈ 2), and Area 51 cells in a single-gene regulatory circuit. (*a*) Steady-state values for equation (2.2) without drive or delay (*α*_0_ = 0, *d* = 0). Depending on the initial value *x*(*t* = 0), the somatic (solid line (red)) and the iPS cells (dash-dotted line (blue)) are stable. The unstable state *x* = 1 (dashed line (green)) is also shown. If the initial state *x*(*t* = 0) has a value infinitesimally above the unstable state *x* = 1, the system transitions to the pluripotent state (+ points), while if *x*(*t* = 0) has an infinitesimally smaller value than *x* = 1 the system transitions to the somatic state (× points). (*b*) Corresponding steady states with a non-zero drive (*α*_0_ = 0.5), a decay constant *β* = 0.5, and the coefficient of self-promotion *α*_1_ = 1.0. Depending on the duration *d* = 2 (solid line (red)) the somatic, or *d* = 3 (dash-dotted line (blue)) iPS cells are chosen. (*c*) Shows *x*(*t*) versus *t* corresponding to equation (2.2) for a delay of *τ* = 500 and for drive *d* = 10 (solid line (red)), and *d* = 1000 (dashed line (blue)) indicating stability of somatic and iPS states. (*d*) Shows *x*(*t*) versus *t* for *d* = 500 with sustained fluctuations between the iPS and somatic states. (Online version in colour.)

We first introduce a single-variable model for cell differentiation, where a single regulator *x* self-regulates its own synthesis, as proposed by Ferrell [15–17]. The equations governing the rate of change of expression of a single gene is given by2.1
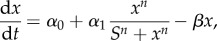
where the first term represents an external input *α*_0_ that is constantly applied. The second term represents a feedback-dependent self-regulation, modelled by a Hill function of order *n*. The third term models degradation process through a mass action process with the degradation rate *β*. The right-hand side of equation (2.1) can be integrated with respect to the variable *x* to give an ‘effective potential’ landscape having two stable minima corresponding to different levels of expression of the gene. This can be seen in figure 1*a*. The two stable fixed points correspond to 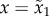 and 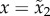, respectively (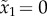 and 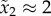 for *α*_0_ = 0) with an unstable extremum at *x* = x* (x* = 1, for *α*_0_ = 0). In the absence of drive, the final gene expression level is crucially dependent on its initial value *x*(*t* = 0). Therefore, if 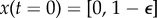 the system approaches 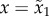, while if 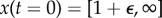, the fixed point 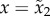 is chosen. Furthermore, in this model beyond a critical value of the external input (*α*_0_ > *α*_c_), the minimum at 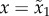 becomes unstable and the long-time steady state is always 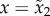. This is in line with Ferrell's idea that saddle-node bifurcations are inconsistent with Waddington's landscape picture as there are no alternative endpoint states. In his work, Ferrell [15–17] further introduces a two variable gene regulatory circuit as a model mimicking lateral inhibition and demonstrates pitchfork bifurcation commensurate with Waddington's picture. A similar two variable model had been proposed around the same time by Wang *et al.* [14].

Motivated by these gene regulatory network models that attempt at developing a quantitative picture of Waddington's landscape, we propose a simple generic single-gene regulatory network model similar to Ferrell [15–17] incorporating time-dependent drive and delay. The rate of change of the gene regulator *x* in this model is described by2.2
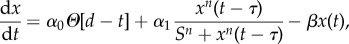
where *α*_0_, *α*_1_ and *β* have the same meanings as equation (2.1). However, unlike that model both the chemical drive as well as the feedback is functions of time. The Heaviside function multiplying the *α*_0_ term represents the fact that the external input is applied for a finite time-interval *d*, while the self-regulatory term is dependent on the state of the regulator *x* at a previous instant of time *t* − *τ*. The time delay in the self-regulation term in equation (2.2) can have several possible physical origins, including multi-step chemical reactions and cell shape changes. We have assumed no such delay in the degradation term, as it does not have biochemical warrant at the same level as the self-regulation and it does not affect the general results in our model.

We numerically integrated equation (2.2) for different values of the delay time *τ* and drive *d*. Figure 1*b* represents the results of the single-gene regulatory circuit without delay and with a chemical drive acting for a finite interval *d* on an initial state *x* = 0. The self-promotion rate coefficient is *α*_1_ = 1 and the decay constant *β* = 0.5. Unless otherwise specified the exponent in the self-regulatory term is chosen to be *n* = 5. Further, the amplitude of the chemical drive is parametrized by *α*_0_ = 0.5. We find that for a value of *α*_0_ < *α*_c_ and the duration of the drive *d* less than a critical value *d*_c_(≈2), the long-time steady state is *x* = 0. If however the drive is applied for a duration longer than *d*_c_, starting from a state *x*(*t* = 0) = 0 the system transitions to the other minimum *x* ≈ 2. Identifying the *x* = 0 state as a somatic and *x* ≈ 2 as the pluripotent state, the above process describes inducing pluripotency via a chemical drive.

Figure 1*c* shows the variation of *x*(*t*) versus *t* starting from the somatic state *x* = 0 for *d* = 10 and *d* = 1000, and a time delay *τ* = 500 for the same set of parameters *α*_0_, *α*_1_ and *β*. As seen in the figure for *d* = 10, the system relaxes back to the *x* = 0 steady state, while for *d* = 1000 the pluripotent state *x* ≈ 2 is chosen. Sharp spikes showing attempted transitions between the two states are also seen. In the intermediate regime when the drive *d* is of the same order of magnitude as the delay *τ*, the trajectory of *x*(*t*) shows sustained oscillations (this is shown in figure 1*d*). We interpret such sustained oscillations as the cells which are caught in a limbo between the pluripotent and the somatic states and conjecture that these states are possibly the ones seen in the experiments by Nagy & Nagy [10] termed ‘Area 51’. The chemical drive *α*_0_ is then interpreted as the doxycycline input to somatic cells having a non-zero value, corresponding to a finite rate of basal synthesis, which is switched off (*α*_0_ = 0) beyond the input time.

**Figure 2.**
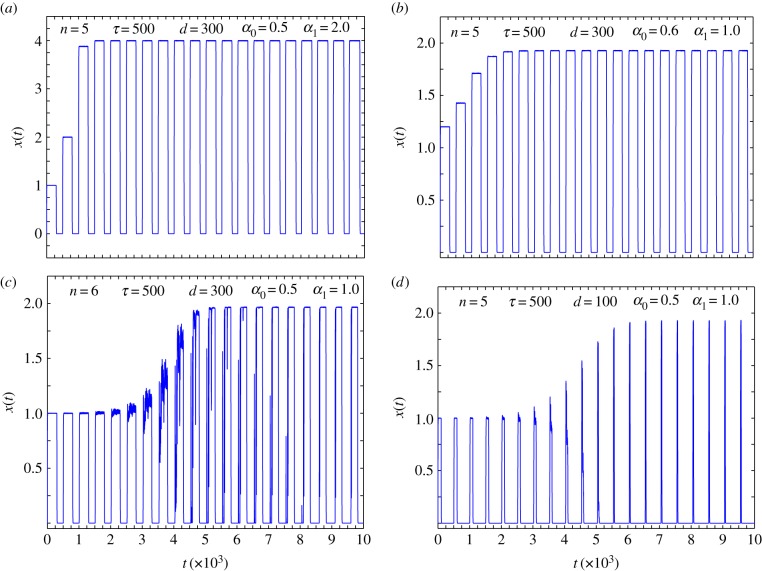
‘Area 51’ oscillations as a function of parameters. The presence of the oscillatory state for different values of the parameters *α*_0_, *α*_1_, that characterize the single-gene expression kinetics, the driving time *d*, delay time *τ* and the order of the Hill function *n*. (*a*) Presence of ‘Area 51’ states for parameter values *n* = 5, *τ* = 500, *d* = 300, *α*_0_ = 0.5 and *α*_1_ = 2.0. Changing the parameters *α*_0_ = 0.6 and *α*_1_ = 1.0, while keeping *n*, *τ* and *d* unchanged also shows oscillations as in (*b*). For a choice of parameters *α*_0_ = 0.5, *α*_1_ = 1.0, and *n* = 6 while keeping parameters *τ* and *d* same as (*a*) shows oscillations with accessible short lived intermediate states that lie between pluripotent and somatic fixed points. This is shown in (*c*). In (*d*) by changing the driving time to a lower value *d* = 100 and *n* = 5 while holding all other parameters same as in (*c*) the duration of time spent in the somatic state can be increased. (Online version in colour.)

The oscillations seen in some solutions of equation (2.2) are an inherent feature of delay differential equations [26]. Sustained oscillations are present for other choices of the model parameters, *α*_0_, *α*_1_ and the order of the Hill functions *n* that characterize the chemical kinetics of gene regulatory circuit. This is shown in figure 2 in which we illustrate the presence of the oscillatory state for different choices of the various parameters. These model parameters encapsulate the underlying biological mechanisms which accompany epigenetic changes. These parameters are thus to be used as inputs from experiments, or detailed molecular-level simulations. As is apparent from the different panels of figure 2, the relative time spent in the somatic and pluripotent state in the oscillatory regime is determined by the precise value of the driving time, and the inherent time delay of the gene network. The sustained oscillations are expected to be biologically relevant when the relative time spent in the two states is of the same magnitude, and this regime is obtained when the drive time *d* is less than the delay time *τ* (*d* ≈ 300 for *τ* = 500, for our choice of parameters), which is a reasonable assumption for a real biological system. The theoretical model maps the full phase diagram, and real-life experiments can then help identify which region of the phase space is occupied by a biological system.

**Figure 3.**
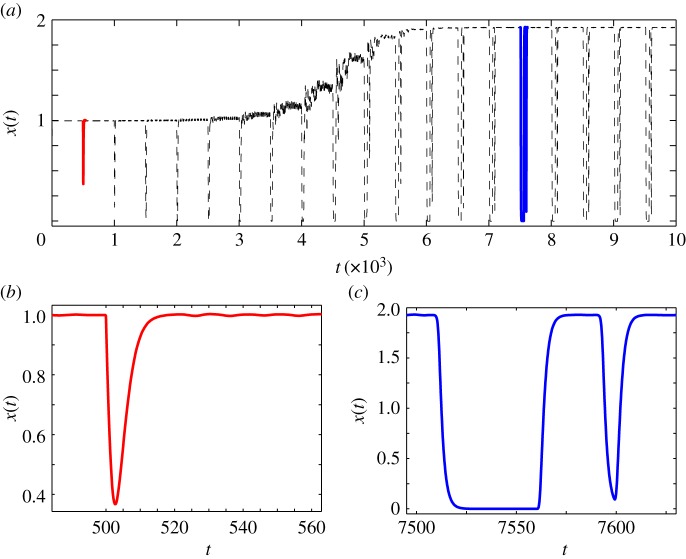
Intermediate states in cellular reprogramming. Fluctuations in the ‘Area 51’ region as a combined result of time-dependent drive *d* and delay *τ* for *d* = *τ* = 500. (*a*) Sustained oscillations for the parameters of figure 1*d*. (*b*,*c*) Indicate the oscillations in the transient (500 ≤ *t* ≤ 540) and sustained oscillatory (7500 ≤ *t* ≤ 7650) regions. (Online version in colour.)

The oscillations as shown in figure 1*d* are investigated in greater detail in figure 3 for *d* = *τ* = 500, and *α*_0_ = 0.5, *α*_1_ = 1 and *n* = 5. It is possible to analyse the time of occurrence of these sharp spikes. If the drive duration is smaller than the delay time, i.e. *d* < *τ*, *x* initially increases from its zero value as a function of time. Once the drive is withdrawn the dynamics of the system is completely dominated by the degradation term and as a result *x* decreases. This behaviour continues till *t* = *τ* when the self-regulation term promoting gene activity becomes non-zero, and as a result *x* increases monotonically till a time *d* + *τ*. At this time, the self-regulatory term picks up the values of *x* from the earlier cycle which was dominated by degradation kinetics. This can be generalized to state that the downward spikes occur at *t_p_* = *d* + *pτ*, while the upturns occur at *t* = *qτ*. The slope of the first downturn is completely dictated by *β* while the upturn slope turns out to be a nonlinear function of *α*_1_ and *β*. For the situation in which *d* > *τ* the first upward turn occurs at *t* = *τ* followed by a downturn upon reduction of the drive at *t* = *d* + *τ*. Following this, oscillations are repeated at *t* = *t_p_* as discussed above. The preceding analysis is strictly valid in the initial time regime, where the spikes occur singly, as shown in figure 3*b*. At later times, the single spikes give way to a double spike, with two spikes occurring in quick succession, as shown in figure 3*c*. A complete description of the behaviour of the oscillations in this later time regime requires a full nonlinear analysis of the original equation.

The two critical timescales alluded to earlier, the ‘PNR’ and ‘CPS’, are shown in figure 1*c* and *d*, respectively. These indicate threshold values such that for *d* < *d*_PNR_ the system would return to their somatic state, while for *d* > *d*_PNR_ the cell fate is changed. The second threshold corresponds to the drive being on for a duration *d* > *d*_CPS_ which results in a final pluripotent cellular state. The intermediate region of drives *d*_PNR_ < *d* < *d*_CPS_ defines the ‘Area 51’ region. Taking cue from our numerical results discussed above, we draw a phase diagram showing the domain of ‘Area 51’ as functions of *d* and *τ* in a single-gene regulatory circuit incorporating time-dependent drive and delay dynamics.

**Figure 4.**
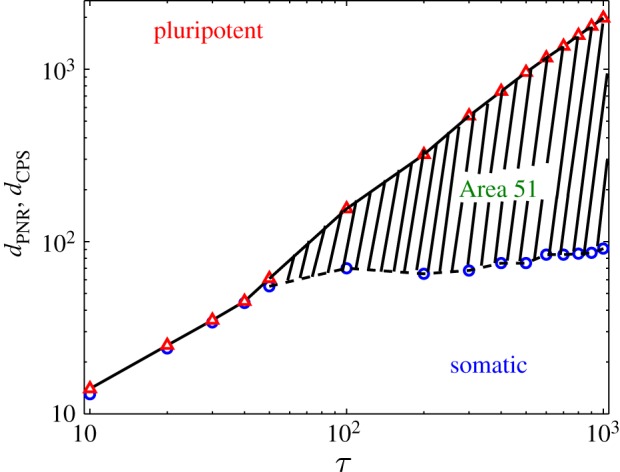
Phase diagram showing regions where somatic and pluripotent states are stable as a function of the delay time *τ*. The phase boundaries indicating point of no return (circles (blue) and dashed line), *d*_PNR_, and those committed to the pluripotent state (triangles (red) and solid line), *d*_CPS_ are indicated. The region between the two states marks the region when the cell fate attains neither fixed point, but oscillates indefinitely, termed ‘Area 51’ [10]. (Online version in colour.)

**Figure 5.**
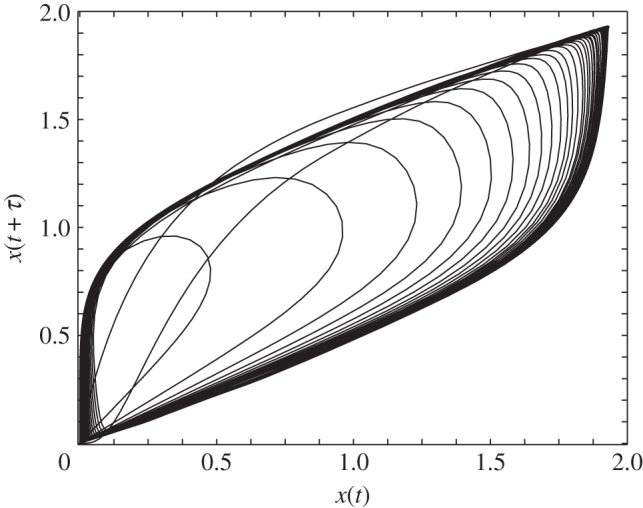
Phase diagram of the expression level of the gene *x*(*t*) as a function of its delayed response *x*(*t* + *τ*). Limit cycle behaviour for the genetic circuit for delay parameters *d* = 500 for the chemical drive and *τ* = 500 for the positive chemical feedback in equation (2.2).

Figure 4 demonstrates the variation of the two thresholds *d*_PNR_ and *d*_CPS_ as a function of the delay *τ*. For 0 ≤ *τ* ≤ 50, the two threshold values are almost the same, i.e. *d*_PNR_ ≈ *d*_CPS_. In this regime, the system transitions from the somatic state to the induced pluripotent state once the duration of the drive is greater than *d*_PNR_. However for larger values of *τ*, the two threshold values are different exposing an intermediate regime marked by sustained oscillations. As seen from the graph, *d*_CPS_ monotonically increases with delay *τ* while some fluctuations in *d*_PNR_ are observed. With increasing *τ*, the ‘Area 51’ region widens as can be seen in figure 4. Phase plots revealing the single-gene expression level for the regulatory circuit is shown in figure 5 in which *x*(*t*) is plotted against *x*(*t* + *τ*). The drive is provided for a time *d* = 500 and the time-delay parameter *τ* = 500. These parameters correspond to the shaded region of figure 4, i.e. ‘Area 51’. A limit cycle is observed indicating the presence of sustained nonlinear oscillations.

## 3. Discussion

We have illustrated the importance of time delays in feedback circuits in the context of a simple gene regulatory network, in which the state of differentiation is regulated by a single differential regulator. The energy landscape of the model, in the absence of delays, has two minimas, denoting the pluripotent and differentiated states. Introducing a delayed self-regulation term changes the landscape such that there is now a region in phase space, in which the system shows sustained oscillations (figures 2 and 3*a*) and the steady state corresponds to a limit cycle (figure 5). We propose that such oscillatory states may underlie the existence of novel intermediate states observed in the reprogramming of mouse somatic cells, and denoted by ‘Area 51’. We hope that our prediction of a long-lived intermediate oscillatory state will motivate future experiments on studying the reprogramming pathways of the cellular differentiation process. Experiments with fast decaying reporters which are proxies for pluripotency or somatic cell markers may provide one avenue for exploring the predicted oscillatory state. If the oscillatory state is experimentally validated, this would then help identify which markers of pluripotency are responsible for the oscillations. This will give a better understanding of the delay timescale, and help identify the regime of parameter space which is appropriate for analysing a real biological system.

In order to model more realistic differentiation events, one would need to study higher dimensional systems, where the number of differential regulators is more than one. Two variable gene regulatory models [14] offer a straight forward generalization of these ideas to mimic realistic cell differentiation scenarios. For a full description of the dynamics of the reprogrammed cell due to the four Yamanaka factors, one needs to study the effect of delays in a four variable model, and map out the effect of the interplay of these four variables on the intermediate state.

The switch from the somatic state to the pluripotent state is accompanied by various changes inside the cell, including changes in the chromatin structure, loss of somatic cell-specific markers, and reactivation of endogenous genes essential for pluripotency and self-renewal, among others. Recent experiments suggest that the various changes associated with pluripotency occur in a well-defined sequential manner. For instance, the pluripotency marker of mouse pluripotent cells, SSEA-1 appears to be expressed in the very early stages of pluripotency [33,34], while the reactivation of endogenous genes such as Oct4, Nanog and Sox2 occurs late in the reprogramming process. It is probable that the rapid fluctuations predicted by the delayed self-regulation model proposed here arise only in the context of one or a few of these pluripotency markers, instead of the full state of the cell switching from somatic to pluripotent. Thus, experiments designed to validate this hypothesis of a fluctuating intermediate state need to identify the probable candidates for such switching.

Another area of interest in the context of induced pluripotent cells is whether there is an inherent asymmetry to the landscape. Nagy *et al.* do not comment whether the ‘Area 51’ is encountered if we perform the reverse experiment, i.e. start from the pluripotent state and induce differentiation by keeping the cells in a chemical environment for different durations. Further experiments are needed to map out the landscape as a pluripotent cell divides under the influence of time-dependent stimuli. Such experiments would then provide an additional input to the model to facilitate understanding of the full epigenetic landscape.

The concept of time delays, possibly induced by remodelling of cellular architecture, is an important one in the differentiation context, as reorganization events inside the cell that accompany a change in cell state take place over a timescale of days [35]. Thus when modelling the epigenetic landscape through dynamical equations, one must consider the effect of delays on differentiation pathways. Similar oscillatory behaviour has also been observed in other related biological systems, such as the epithelial to mesenchymal transition in early embryonic development and cancer metastasis [36–38]. In both these situations, the oscillations arise from time-dependent remodelling of the cytoskeleton. Thus, the concept of delays may be important also in other biological contexts and should prove a useful tool in the design of predictive experiments.

It is natural to ask the question whether there exists an equivalent ‘Area 51’ intermediate state when the more common experimental scenario, i.e. studying cell differentiation starting from an initial pluripotent state is considered. Such an experiment would involve withdrawing the chemical drive responsible for differentiation at different stages of development. The original experiments of Nagy *et al.* do not shed light on this scenario.

Our mathematical model is constructed such that the somatic state is identified as 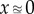 while 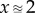 is the pluripotent one. Application of a chemical drive (*α*_0_ > 0) with the initial state at 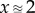 (pluripotent) will not result in an ‘Area 51’ or even differentiation to a somatic state.

However, if we hypothesize that starting from the pluripotent state, *α*_0_ < 0, corresponds to the reverse situation, i.e. the presence of a morphogen that induces differentiation, then for a choice of parameters *d*, and *τ* transition to the pluripotent state as well as an intermediate state characterized by sustained fluctuations is obtained.

This opens up the interesting question of addressing which model correctly describes the physical scenario, a delayed model versus a tri-stable chemical reaction system with the three minima corresponding to somatic, pluripotent and Area 51. In such a chemical reaction system, as the chemical drive (in the form of a linear potential ramp) is applied over a period of time, the somatic, intermediate Area 51 and pluripotent minimas would become unstable in a similar manner as our delayed model. However, the precise nature of bifurcations and phase transitions that would arise in these two different systems would be different. Further theoretical work involving a full nonlinear analysis of the model backed up by careful experimentation would be required to discern between these two scenarios. We hope that our work will prompt such experiments.
